# Molecular profiling of core immune-escape genes highlights LCK as an immune-related prognostic biomarker in melanoma

**DOI:** 10.3389/fimmu.2022.1024931

**Published:** 2022-10-20

**Authors:** Fang Wang, Anfu Zheng, Duoli Zhang, Tao Zou, Mintao Xiao, Jie Chen, Bo Wen, Qinglian Wen, Xu Wu, Mingxing Li, Fukuan Du, Yu Chen, Yueshui Zhao, Jing Shen, Shixin Xiang, Jing Li, Shuai Deng, Zhuo Zhang, Tao Yi, Zhangang Xiao

**Affiliations:** ^1^ Laboratory of Molecular Pharmacology, Department of Pharmacology, School of Pharmacy, Southwest Medical University, Luzhou, China; ^2^ Department of Medical Technology, Faculty of Associated Medical Sciences, Chiang Mai University, Chiang Mai, Thailand; ^3^ Department of Oncology, Affiliated Hospital of Southwest Medical University, Luzhou, Sichuan, China; ^4^ Cell Therapy & Cell Drugs of Luzhou Key Laboratory, Luzhou, Sichuan, China; ^5^ South Sichuan Institute of Translational Medicine, Luzhou, Sichuan, China; ^6^ Department of Pharmacy, University-Town Hospital of Chongqing Medical University, Chongqing, China; ^7^ Department of Oncology and Hematology, The Affiliated Traditional Chinese Medicine (TCM) Hospital of Southwest Medical University, Luzhou, China; ^8^ School of Chinese Medicine, Hong Kong Baptist University, Hong Kong, Hong Kong SAR, China

**Keywords:** melanoma, single-cell sequence, tme, oncoPredict, immune escape

## Abstract

The tumor microenvironment is complicated and continuously evolving. This study was devoted to the identification of potential prognostic biomarkers based on the tumor microenvironment associated with immunotherapy for melanoma. This study integrates a couple of melanoma single cell and transcriptome sequencing datasets and performs a series of silico analyses as nicely as validation of molecular biology techniques. A core set of immune escape related genes was identified through Lawson et al. and the ImmPort portal. The differential proteins were identified through the cBioPortal database. Regression analysis was used to profile independent prognostic factors. Correlation with the level of immune cell infiltration was evaluated by multiple algorithms. The capacity of LCK to predict response was assessed in two independent immunotherapy cohorts. High LCK expression is associated with better prognosis, high levels of TILs and better clinical staging. Pathway analysis showed that high expression of LCK was significantly associated with activation of multiple tumor pathways as well as immune-related pathways. LCK expression tends to be higher in immunotherapy-responsive patients and those with lower IC50s treated with chemotherapeutic agents. RT-qPCR detected that LCK expression was significantly upregulated in melanoma cell lines. Single-cell transcriptome analysis showed that LCK was specifically highly expressed on T cells. CellChat analysis confirmed that LCK in C2 subpopulations and T cell subpopulations exerted immune promotion between cells by binding to CD8 receptors. In conclusion, LCK is a reliable biomarker for melanoma and will contribute to its immunotherapy.

## Introduction

Melanoma is commonly located on skin, and its major metastatic sites include mucous membranes and internal organs. Due to its high aggressiveness and dangerousness, melanoma accounts for up to 75% of skin cancer deaths, even though it accounts for only 5% of skin cancers (1). Related data suggest that there will be approximately 98,000 confirmed cases of melanoma and 7,700 melanoma deaths in the United States in 2022 (2). However, according to the World Health Organization, the morbidity and mortality rates of melanoma have shown a decreasing trend every year (3). Treatment options for melanoma include traditional surgery, radiation therapy, chemotherapy, and emerging treatments including immune checkpoint inhibitors and targeted therapies, among others. The widespread use of new therapeutic approaches has largely improved the prognosis of melanoma (4). The extremely high immunogenicity of melanoma and the high mutational load of its genome make it highly susceptible to triggering specific antitumor immune responses (5). Furthermore, melanoma is a classical model for detecting innovative immunotherapies such as checkpoint inhibitors, engineered chimeric antigen receptor T cells, among others (6, 7). Nevertheless, like the suppressive mechanisms that arise in other cancers, melanoma evades detection by the immune system in concert with these mechanisms and eventually leads to metastasis (5).

The immune system plays a crucial role in the development and treatment of cancer. Adaptive immunity can prevent or constrain cancer through immune surveillance, while innate immunity and inflammation often promote tumorigenesis and malignant development of neoplastic cancers (8). Immunotherapy targeting tumor microenvironment (TME) in the human immune system represents a revolutionary approach to cancer treatment, which enhances anti-tumor immunity by stimulating or mobilizing the body’s immune system (9). TME significantly affects the immunotherapeutic response and clinical prognosis of cancer patients (10–12). As an example, it has been noted that cancer patients with high CD8+ T-cell infiltration levels generally have a better prognosis (13, 14). Conversely, poor prognosis in cancer patients is also thought to be associated with the presence of M2-polarized macrophages (15–17). The TME consists of stromal cells, fibroblasts, endothelial cells, and innate and adaptive immune cells (18). Among them, immune cells, cytokines and cell surface molecules constitute the tumor immune microenvironment, which is described as the “seventh hallmark feature” of tumors. The complex regulatory network in the tumor immune microenvironment plays a key role in tumor progression (19, 20). The vast majority of tumor cells express antigens that mediate recognition by host immune cells (21). However, the presence of tumor immune escape phenomenon makes tumor cells exempt from immune elimination. Mechanisms of tumor immune escape include loss of antigenicity and immunogenicity, as well as coordination of immunosuppressive effects in the microenvironment, among others (22). Although immunogenic antigens are better expressed in tumors, the effect of immunodetection is also dependent on the antigen-presenting ability of the peptide-MHC (major histocompatibility complex) complex (22). However, it has been previously shown that the expression of MHC class I molecules is downregulated in approximately 20-60% of common solid malignancies such as melanoma and lung cancer (23). Tumors that lose MHC expression or present with defective antigen presentation are highly susceptible to immune escape (24). CD8+ effector T cells recognize immune cells through antigens presented by MHC I molecules and inhibit tumor progression by inducing cytotoxicity of tumor cells to inhibit tumor progression (25). In recent years, the wide application of bioinformatics techniques in the field of cancer immunotherapy has helped us to explore more deeply the connection between tumor cells and immune cells. Transcriptome, single cell RNA sequencing, and molecular biology are all sturdy bridges to study the tumor microenvironment.

In this study, RNA transcriptome profiles were extracted from the TCGA database and core immune-related genes were identified from previous studies. Independent prognostic factors significantly associated with prognosis were identified by multiple prognostic analysis methods. The relationship between independent prognostic factors and TME was explored by four methods, EPIC (Estimate the Proportion of Immune and Cancer cells), TIMER, quanTiseq and MCPcounter, and the strong association of LCK with immune components of the tumor microenvironment was confirmed. The specific mechanism of action of LCK acting in tumor cells and immune cells was finally confirmed using single-cell RNA sequencing technology and its related analytical methods.

## Materials and methods

### Data collection

A set of melanoma immune escape related genes were extracted from the Lawson et al. (26) (**Supplementary Material 1**). Immune-related genes were extracted from the IMMPORT database (**Supplementary Material 1**). Gene expression profiles, clinical follow-up information, and somatic mutation data were extracted from The Cancer Genome Consortium (TCGA) database through the ‘TCGAbiolinks’ package. In addition, we extracted normal control samples from the Genotype-Tissue Expression Project to compensate for the absence of normal sample controls in the TCGA cohort. Single cell transcriptome data as well as external validation datasets were downloaded from the Gene Expression Omnibus (GEO) and International Cancer Genome Consortium (ICGC) databases (**Table 1**). Duplicate genes are processed by the avereps function of the ‘limma’ package. Processing of gene expression values into Transcripts Per Kilobase of exon model per Million mapped reads (TPM) and normalized by log2. With different batches of GEO datasets we remove the batch effect by using the combat function of the ‘sva’ package.

**Table 1:** Melanoma External DataSets Summary.

DataSets ID	Melanoma Sample Number	GPL Platform	Total Number
microarray			
GSE15605	58	GPL570	
GSE19234	44	GPL570	
GSE22155	22	GPL6947	
GSE3189	45	GPL96	
GSE46517	104	GPL96	
GSE59455	141	GPL8432	
GSE65904	214	GPL10558	
			628
GSE54467	79	GPL6884	
**Single-Cell**	1	GPL18573	79
GSE72056	3	GPL24676	4
GSE186344			
**ICGC**			
SKCM-US	433	NULL	433

### Somatic cell mutation analysis

Somatic mutation data in mutation annotation format (MAF) were visualized through ‘maftools’ R package, which provides a large number of analysis and visualization modules commonly used in cancer genomic studies (27).

### Identification of differentially expressed proteins

cBioPortal provides a Web resource for exploring, visualizing, and analyzing multidimensional cancer genomics data (28). Differentially altered proteins (both unphosphorylated and phosphorylated) associated with immune escape related genes were identified in cBioPortal by the Reverse Phase Protein Array (RPPA) module.

### Protein-protein interaction

Protein-protein interaction network of protein-coding genes constructed by STRING database. Minimum required interaction score 0.4 and disconnected nodes in the network were hide.

### Construction of risk prognostic models

The melanoma patients from TCGA cohort were divided into a training set (TRS) and a testing set (TES). The TRS was used to construct a prognostic risk model of melanoma and the TES was utilized to valid the predictive capability of this model. ICGC-SKCM-US is used as external dataset validation. Risk prognostic models were constructed by univariate cox regression, least absolute shrinkage and selection operator (LASSO) regression analysis, and multivariate cox regression analysis. The risk score for each sample is calculated as follows:


$$\left[Risk\;Score\;\left(each\;patient\right)=\sum_{i}Expression(mRNA_{i})\times Coefficient(mRNA_{i})\right]$$

Exploring the diagnostic value of risk scores for 1-year, 3-year, 5-year, 8-year and 10-year survival status with the ‘pROC’ package.

### Functional enrichment analysis

‘GSVA’ package was used to explore the signaling pathways in which the screened protein-coding genes are involved and the c2.cp.kegg.v7.5.1.entrez.gmt gene sets was used to calculate differences in enrichment scores for pathways in different populations in the tow group.

### Tumor microenvironment analysis

To explore the role of our screened protein-coding genes and the tumor microenvironment, we used ESTIMATE, EPIC, TIMER, quanTiseq and MCPcounter algorithms to calculate the proportion of various immune factors infiltrating the tumor microenvironment and explored the correlation between protein-coding gene expression and immune factor infiltration levels.

### Single cell sequencing analysis

Considering that the number of cells in one single cell dataset is too small, we removed the batch effect and merged GSE72056 and GSE186344 *via* the ‘harmony’ package. The integrated single cell data was then analyzed using the ‘Seurat’ package, including finding highly variable genes, centralization, PCA downscaling, cell clustering, tSNE (t-Distributed Stochastic Neighbor Embedding) and UMAP (Uniform Manifold Approximation and Projection) nonlinear downscaling, finding differential genes, and cell annotation. To investigate cell-to-cell interactions and to determine the mechanisms of communication molecules at single-cell resolution, 8 cell subgroups were studied using the R package ‘CellChat’.

### Drug sensitivity analysis

To explore the potential of the screened protein-coding genes as predictive biomarkers for chemotherapy or immunotherapy, we attempted to assess the correlation between different expression populations of protein-coding genes and responsiveness. ‘oncoPredict’ is an R package for predicting *in vivo* or cancer patient drug response and biomarkers from cell line screening data (29). We extracted immunotherapy cohort (PRJEB23709 and PRJEB25780) from Tumor Immune Dysfunction and Exclusion (TIDE) to explore the potential of protein-coding genes as predictors of immunotherapy response (**Supplementary Material 2**).

### Cell culture

The PIG1 cells (Otwo Biotech, ShenZhen Inc. China), A2058 and SKMEL28 cells (the Chinese Academy of Sciences) were cultured in the Dulbecco’s modified Eagle’s medium (DMEM) (Gibco, Thermo Fisher Scientific, Inc.) medium containing 10% FBS (Gibco; Thermo Fisher Scientific, Inc.) in an incubator at 37˚C, and the air of the incubator consisted of 5% CO2.

### RT-qPCR

The cells were cultivated in a 6-well plate at a density of 40×10^4^ cells per well and incubated at 37˚C. Following collected the cells after the cell’s density reached 80%. Total RNA was extracted from the 6-well plate using TRIzol reagent (Thermo Fisher Scientific, Inc.), then subjected to reverse transcription-quantitative polymerase chain reaction (RT-qPCR) to detect the mRNA expression of LCK. qPCR was performed with a SYBR Green Real Time PCR kit (Thermo Fisher Scientific, Inc.) on CFX96 Touch Real Time PCR System (BioRad Laboratories, Inc.). The primers used for real-time PCR were at **Table 3** (**Table 2**). qPCR was performed under the following conditions: 3 min at 95˚C for 1 cycle, 10 sec at 95˚C, 30 sec at 65˚C for 39 cycles, and 95˚C for 5 sec. Changes in the expression of target genes were calculated using the 2^-ΔΔCq^ method.

**Table 2:** The premier sequences.

Gene Symbol	Forward	Reverse
LCK	5’- TCTGCACAGCTATGAGCCCT -3′	5’- GAAGGAGCCGTGAGTGTTCC -3′
GAPDH	5’- CTGGGCTACACTGAGCACC -3′	5’- AAGTGGTCGTTGAGGGCAATG -3′

## Results

### Landscape of immune escape related genes in melanoma

**Figure 1:**
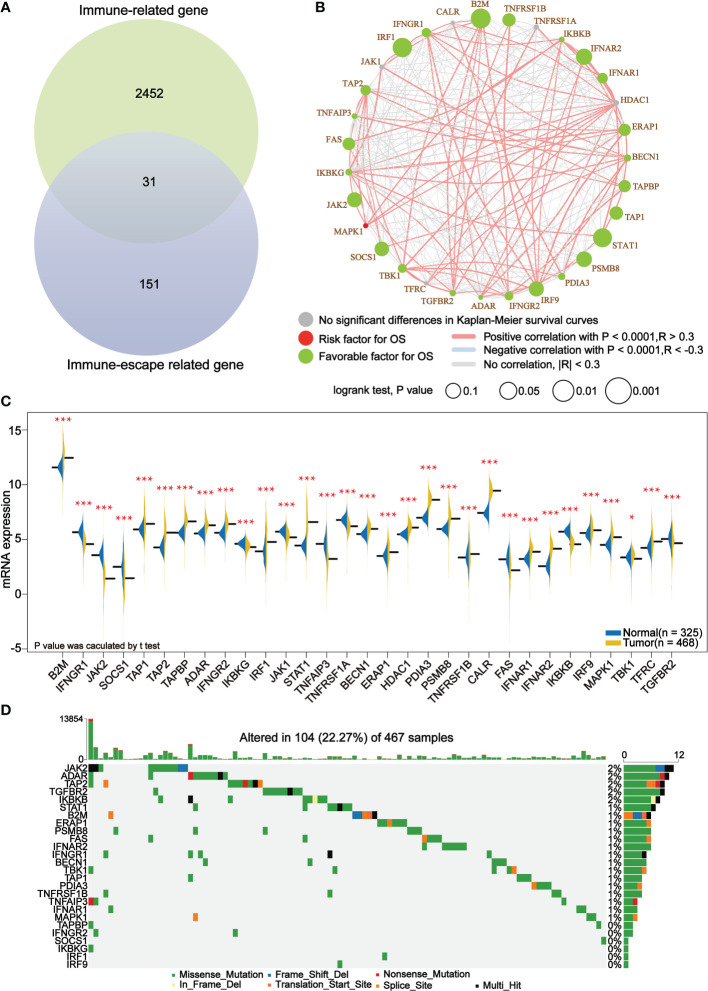
Core immune escape related genes are favorable prognostic factors for melanoma and are significantly upregulated in tumor tissue. **(A)** 31 core immune-escape related genes were identified as shown in Venn diagram. **(B)** Prognostic analysis of core immune-escape related genes. Green dots represent favorable prognostic factors, red dots represent unfavorable prognostic factors, and grey dots represent genes that are not significantly different in the Kaplan-Meier survival curve. The bigger the dot is, the smaller the P value is. The line between the different dots represents the correlation between them. The red line indicates a positive correlation between them, the blue line indicates a negative correlation between them; the grey line indicates that the two are not related. P value calculated by log-rank test and the correlation coefficient between the core immune-escape related genes were evaluated using Spearman’s correlation analysis. **(C)** Expression analysis of core immune escape-related genes. Blue and yellow half-violins represented normal and tumor samples, respectively. **(D)** Waterfall plot of detailed mutation information of 26 genes after initial screening in each sample, with various color annotations to distinguish different mutation types. *P < 0.05, ***P < 0.001.

**Figure 2:**
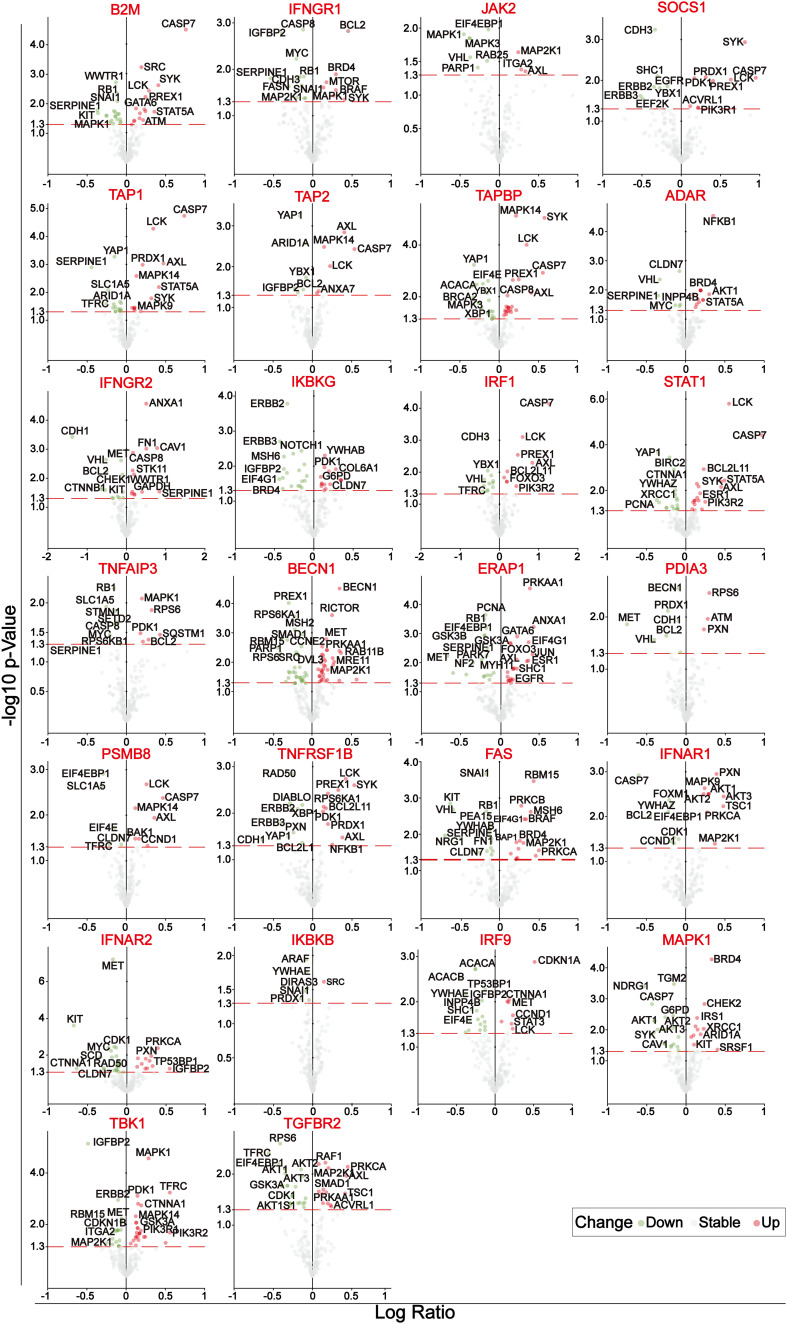
Volcano plots of the differentially expressed proteins of 26 genes after initial screening. The circles with green, gray and blue represent significantly down-regulated, no significant change and significantly up-regulated differentially expressed proteins, respectively.

A set of immune-escape related genes and a set of immune-related genes were extracted from the previous study and from the IMMPORT website respectively, which contains 182 genes and 2483 genes respectively in total. Overlapping these two gene sets, 31 core immune-escape related genes (**Table 3**) were determined as the subjects for further analysis (**Figure 1A**). In addition, 472 (including 471 tumor samples and 1 normal sample) and 398 normal samples were obtained from the TCGA and GTEx databases, respectively. Prognostic Analysis showed that except CALR, TNFRSF1A, HDAC1, JAK1 and TFRC, which were not significantly different, and MAPK1, which was an unfavorable prognostic factor, the rest of all the core immune-escape genes were favorable prognostic factors, and all of them were significantly different (**Figure 1B**). Expression analysis showed that the expression of all core immune escape related genes was dysregulated. IFNGR1, JAK2, SOCS1, IKBKG, JAK1, TNFAIP3, TNFRSF1A, FAS, IKBKG, TBK1 and TGFBR2 were highly expressed in normal tissues, while the expression of the remaining genes were upregulated in tumor tissues, all of the above differential expression analysis results were significantly different (p-value< 0.05) (**Figure 1C**). Genes with significantly different in both expression difference analysis and prognostic analysis were initially screened out for inclusion in the next analysis. Somatic mutation profiles of 467 melanoma patients downloaded from TCGA database were analyzed and visualized *via* the ‘maftools’ R package (30). The results showed that they all had low mutation rates (**Figure 1D**). In addition, identification of differentially expressed proteins regulated by primary screening genes through the RPPA module of the cBioPortal database and 164 differentially expressed proteins were obtained in total (**Figure 2**).

**Table 3:** A list of core immune-escape related genes.

Gene Symbol
ADAR, B2M, BECN1, CALR, ERAP1, FAS, HDAC1, IFNAR1, IFNAR2, IRF1, IRF9, IFNGR1, IFNGR2, IKBKG, IKBKB, JAK1, JAK2, MAPK1, PDLA3, PSMB8, SOCS1, STAT1, TAP1, TAP2, TAPBP, TBK1, TFRC, TGFBR2, TNFAIP3, TNFRSF1A, TNFRSF1B

### Assessment of risk characteristics associated with prognosis of melanoma

**Figure 3:**
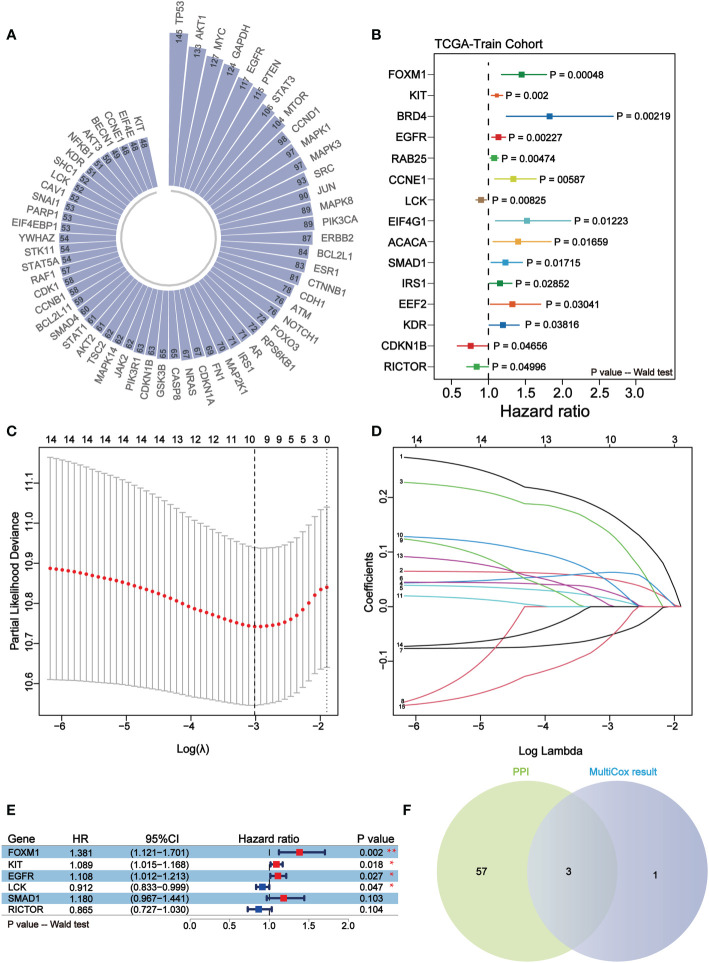
KIT, EGFR and LCK may serve as key independent prognostic factors for melanoma. **(A)** Circular bar plot of the top 60 nodes in the protein-protein interaction network in terms of connectivity. **(B)** Forest plot of the results of univariate COX regression analysis of 164 differentially expressed proteins. Different colors indicate differentially expressed proteins. **(C, D)** The LASSO regression analysis for the 15 prognostic factors. The coefficient profile plot **(D)** was generated against the log (lambda) sequence **(C)**. **(E)** Forest plots of 6 independent prognostic factors in multivariate COX regression analysis. Red represents unfavorable prognosis and blue represents favorable prognosis. **(F)** Venn diagram of the results of the overlapping protein-protein interaction network analysis and multifactor COX regression analysis. 3 key independent prognostic factors were identified, namely KIT, EGFR, and LCK.

Through STRING database, protein-protein interaction network was constructed based on the above differentially expressed proteins, including 188 nodes and 3670 edges in summary. Subsequently, we filtered out the top 60 nodes in the entire network in terms of connectivity (**Figure 3A**). Meanwhile, the melanoma samples were divided into TRS and TES in a roughly 1:1 ratio (228 samples in TRS and 226 samples in TES). The result of univariate cox regression analysis of TRS showed that a total of 15 prognostic factors were determined. Except CDKN1B, LCK, and RICTOR which were favorable prognostic factors, the rest of these prognostic factors were associated with reduced overall survival (p-value< 0.05) (**Figure 3B**). Immediately, LASSO regression analysis of prognostic factors was performed and 10 representative protein-coding genes were identified (**Figures 3C, D**, **Table 4**). Multivariate cox regression analysis was conducted within these 10 representative protein-coding genes and 4 independent prognostic factors were finally identified which were related to prognosis in melanoma (**Figure 3E**). Overall, all 4 independent prognostic factors were associated with reduced overall survival, except for LCK, which was favorable factor (**Figure 3E**). Overlapping the top 60 nodes in the protein-protein interaction network in terms of connectivity and the 4 significant independent prognostic factors, we obtained 3 key prognostic factors, respectively KIT, EGFR and LCK (**Figure 3F**).

**Table 4:** The results of LASSO regression analysis.

Gene Symbol
FOXM1, KIT, BRD4, EGFR, RAB25, CCNE1, LCK, SMAD1, KDR, RICTOR

### Risk-prognosis models constructed by independent prognostic factors may prolong overall survival of melanoma

**Figure 4:**
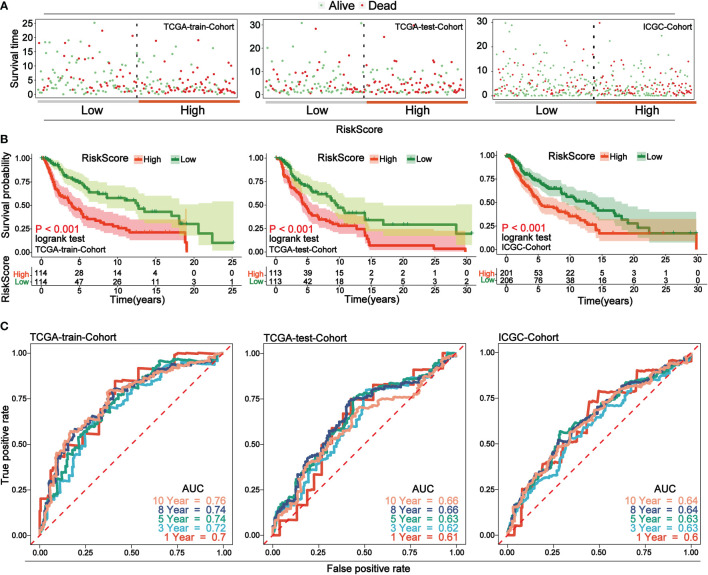
Low-risk group in prognostic risk model associated with longer overall survival. **(A)** Risk factor analysis of the three cohorts, with samples divided into two groups of high and low risk based on risk scores, with green dots representing surviving samples and red dots representing dead samples. As the risk score increases, the number of dead samples increases. **(B)** Survival curves for high and low risk scores for the three cohorts. In all cohorts, low-risk scores were associated with improved overall survival. **(C)** Time-dependent ROC curves for the three cohorts. Curves with different colors indicate different time points. The AUC values above 0.6 at different time nodes demonstrate the accuracy of the prognostic model.

Through analyzing the independent prognostic factors in TRS, a risk prognostic model was constructed in TRS, using which the prognosis of patients could be effectively predicted. To verify the predictive efficacy of the model, the same model was constructed in TES and the ICGC cohort. The results of the risk factor analysis showed that the number of patient deaths clustered significantly as the risk score increased (**Figure 4A**). Survival analysis in the high- and low-risk score groups showed that the high-risk group was associated with lower overall survival (p-value< 0.05) (**Figure 4B**). Finally, time-independent ROC curves were established to verify the accuracy of the model. The AUC values of the ROC curves for the three cohorts indicate that the model has good accuracy (**Figure 4C**).

### LCK is a promising indicator for remodeling the tumor microenvironment of melanoma

**Figure 5:**
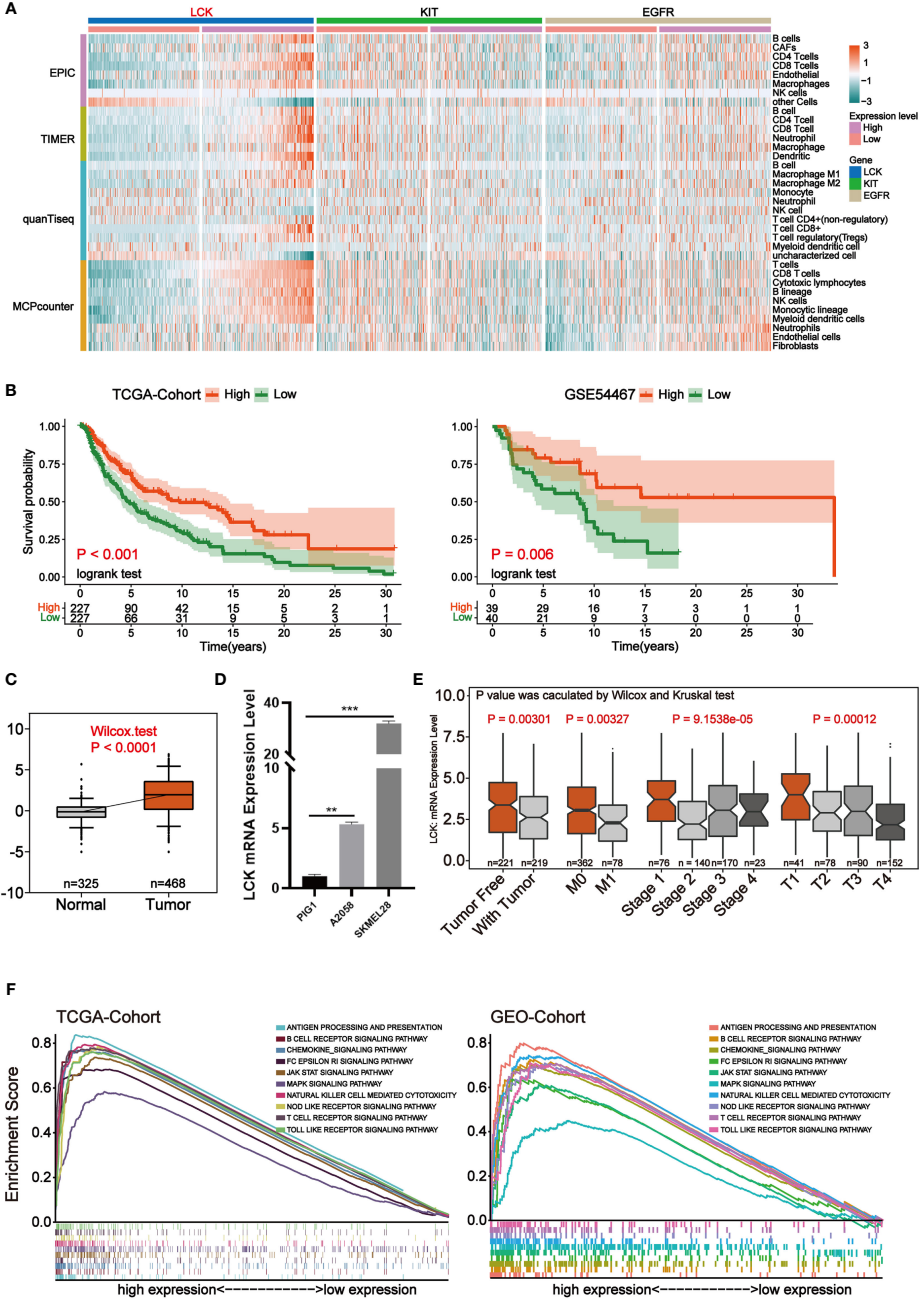
LCK is the most critical independent prognostic factor that may reshape the tumor microenvironment. **(A)** Heat map of the level of immune cell infiltration for each sample of melanoma. The four modules in the rows represent the level of immune cell infiltration calculated by the four algorithms. Columns represent samples of melanoma under three genetic groupings. The heat map from blue to red indicates the infiltration level from low to high. **(B)** Kaplan-Meier survival analysis for high and low expression levels of LCK in the TCGA cohort and the GSE54467 cohort. **(C)** Differential expression analysis of LCK in tumor and normal samples. **(D)** RT-qPCR of LCK in PIG1, A2058 and SKMEL28 cell lines. **(E)** Box plot of the correlation of LCK expression levels with clinical parameters. **(F)** GSEA was performed based on LCK high and low expression in the TCGA cohort and the integrated GEO cohort. **P < 0.01, ***P < 0.001.

The infiltration level of immune cell was calculated for each sample of melanoma using four methods, EPIC, TIMER, quanTiseq and MCPcounter, respectively. Focusing on the three key independent prognostic factors mentioned above, we found that samples with high LCK expression were accompanied by higher levels of immune infiltration (**Figure 5A**). Therefore, we suggest that LCK may affect prognosis by altering the tumor microenvironment of melanoma and thereby. Survival analysis in both the TCGA cohort and the GSE54467 cohort of melanoma showed that the group with high LCK expression levels was associated with prolonged overall survival (p-value< 0.05) (**Figure 5B**). In normal and tumor tissues, differential expression analysis based on TCGA cohort and GTEX cohort showed that LCK was highly expressed in tumor tissues (p-value< 0.05) (**Figure 5C**). To verify the expression pattern of LCK, RT-qPCR was used to detect the mRNA expression of LCK. The results showed that the expression level of LCK was significantly higher in melanoma cell lines (A2058, SKMEL28) than in normal skin cell lines (PIG1, p-value< 0.05) (**Figure 5D**). We then analyzed the correlation between LCK expression levels and four clinical parameters, including tumor status, metastasis, pathological stage, and the extent and size of the primary tumor. The results indicated that the expression level of LCK was significantly downregulated as melanoma progressed clinically (p-value< 0.05) (**Figure 5E**). Subsequently, gene set enrichment analyses (GSEA) were carried out in the TCGA cohort and the GEO cohort. The GEO cohort we use here was integrated from 7 separate data, including GSE15605, GSE19234, GSE22155, GSE3189, GSE46517, GSE59455 and GSE65904 (**Supplementary Figure 1**). The results of the enrichment analysis showed that the gene set in the LCK high expression group was significantly enriched in cancer progression-related pathways and immune component-related pathways (**Figure 5F**). The above results illustrate that LCK plays an important role in remodeling the tumor microenvironment of melanoma.

### In melanoma, LCK is highly correlated with immunity and can predict response to immunotherapy

**Figure 6:**
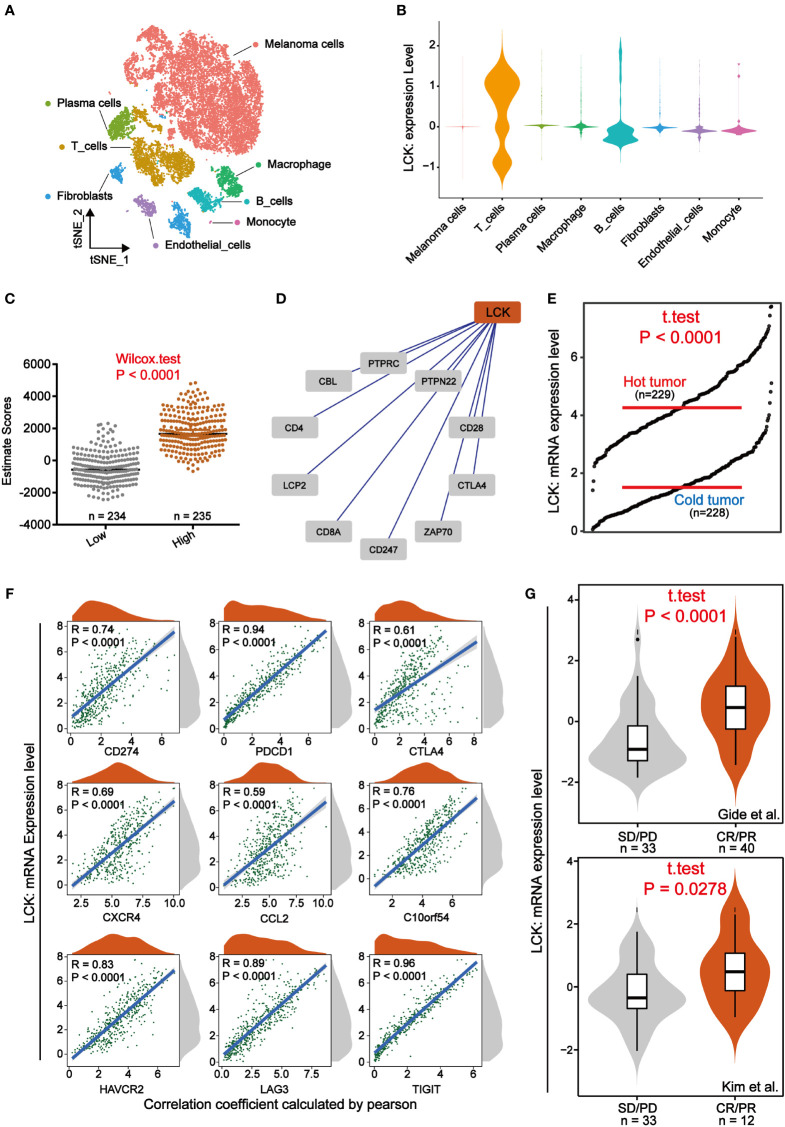
LCK is highly correlated with immunity and can predict response to immunotherapy. **(A)** A tSNE view of 18690 single cells, colour-coded by assigned cell type. **(B)** Violin plots of the expression levels of LCK in different cell types. LCK was specifically expressed in T cells. **(C)** Scatter plot of ESTIMATE scores in LCK high and low expression subgroups. The ESTIMATE scores were higher in the group with high LCK expression levels. **(D)** Protein-protein interaction network plot constructed based on LCK. **(E)** The grdotplot of LCK expression levels in melanoma samples under cold and hot tumor groupings. **(F)** Scatter density plot of the correlation analysis between the expression levels of LCK and the expression levels of the 9 immune checkpoints. The expression level of LCK showed a significant positive correlation with the expression level of immune checkpoints. **(G)** Violin plot of the differential expression analysis of LCK in two immunotherapy cohorts. CR/PR and SD/PD represent the response and non-response groups to immunotherapy, respectively.

To explore the specific role played by LCK in the tumor microenvironment of melanoma, we analyzed the immune landscape of LCK in melanoma. To begin, we focused on single-cell sequencing data from melanoma. We integrated single-cell sequencing data from two melanoma cases and further completed the dimensional reduction clustering and annotation (**Supplementary Figures 2A–D**, **Figure 6A**). We then found that LCK was significantly highly expressed in T cells and could be further used as a marker for T cells (**Figure 6B**). Subsequently, we calculated the ESTIMATE score for each sample in the TCGA cohort through the ESTIMATE algorithm. We further found that ESTIMATE scores were significantly higher in the group with high LCK expression levels than in the group with low LCK expression levels (p-value< 0.05) (**Figure 6C**). Protein-protein interaction network constructed based on LCK showed that LCK plays a key role in the T-cell antigen receptor-linked signal transduction pathway (**Figure 6D**). Immediately after, we classified the samples in the TCGA cohort of melanoma into cold and hot tumors based on the 12 genes extracted from the study of Chunyu Dong et al. (**Supplementary Figure 3**). We found that LCK expression levels were higher in hot tumors (**Figure 6E**).

Immunotherapy such as immune checkpoint inhibitors have wide application in some solid tumors such as melanoma. However, the issue of patient responsiveness is an obstacle to their effective application. Here, we analyzed the relationship between the expression levels of LCK and the expression levels of the 9 immune checkpoints. The correlation analysis showed that LCK showed a significant positive correlation with immune checkpoints (**Figure 6F**). That is to say, patients with high LCK expression tend to have better immunotherapeutic responses due to high levels of ICPs [31]. Thus, to verify the relationship between LCK and immunotherapy response, we examined the expression levels of LCK in the immunotherapy cohort. Based on 2 immunotherapy cohorts extracted from the TIDE database, we observed that LCK expression levels were higher in the immunotherapy response group (**Figure 6G**). Collectively, in melanoma, LCK is closely linked to the immune components of its microenvironment and is effective in predicting immunotherapeutic response.

### LCK promotes T cell activation and suppresses immune escape of melanoma cells by binding to the CD8 receptor

**Figure 7:**
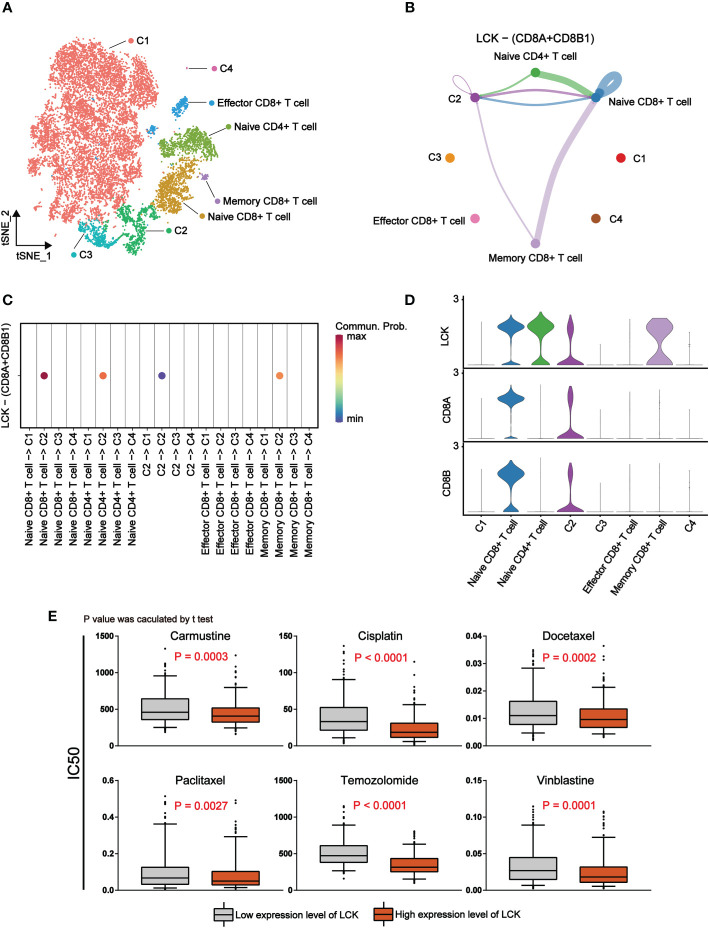
LCK promotes antitumor immune responses by binding to CD8 receptors and is associated with a lower IC50 for antitumor drugs. **(A)** The tSNE plot of 14760 single cells for melanoma cells and T cells, colour-coded by assigned cell type. **(B)** Circle plots of LCK-CD8 receptor interactions in CellChat analysis of melanoma cell subgroups and T cell subgroups. The thickness of the line represents the magnitude of the action intensity; the arrow represents the action direction. **(C)** Bubble plots of LCK-CD8 receptor interactions in CellChat analysis of melanoma cell subgroups and T cell subgroups. **(D)** Violin plots of the expression levels of LCK, CD8A and CD8B in melanoma cell subgroups and T cell subgroups. **(E)** Drug sensitivity analysis based on the expression level of LCK. The IC50 values of the six antitumor drugs were lower in the group with high LCK expression levels.

We found that LCK, a marker of T cells, plays a crucial role in the immunotherapy of melanoma. Thus, we tried to elucidate the specific mechanism by which LCK acts through cellular interactions. Based on the previously annotated single cell sequencing data, we extracted the melanoma cells and T cells separately. By further dimensional reduction clustering, we obtained 14 subgroups (**Supplementary Figures 4A, B**). Based on the differential expression of genes, we classified melanoma cells into 4 categories (**Supplementary Figure 4C**, **Figure 7A**). Meanwhile, we classified T cells into Naive CD4^+^ T cells, Naive CD8^+^ T cells, Effector CD8^+^ T cells and Memory CD8^+^ T cells according to the specific expression of CD4, CD8 (CD8A), CD45 (PTPRC), CD197 (CCR7), CD25 (IL2RA) (**Supplementary Figure 4D**, **Figure 7A**). Subsequently, cell-cell communication was inferred by the ‘CellChat’ package on the basis of the subgroup we annotated. The results showed that there was a strong interaction between melanoma cells and T cells (**Supplementary Figures 4E, F**). Subsequently, we selected LCK-related ligand-receptor pairs left for further analysis. The results suggest that LCK as a ligand acts between the C2 subgroup of melanoma cells and T cell subgroups by binding to the CD8 receptor (**Figures 7B–D**). The binding of LCK to the CD8 receptor drives CD8 close to the TCR and ultimately stabilizes the TCR-MHCp interaction (31). TCR-MHCP interactions promote T cell activation (32). The activation of T cells further increases the response of T cells to pathogens or malignant cells (33). Therefore, we propose that LCK inhibits immune escape of melanoma cells in melanoma by promoting the activation of T cells. Finally, we examined the correlation between the expression levels of LCK and the IC50 of commonly used antitumor drugs. The results showed that the group with high expression of LCK had a lower IC50 (**Figure 7E**). This means that LCK can effectively enhance the inhibitory effect of antitumor drugs.

## Discussion

Although immunotherapy has been used extensively in melanoma, its annual mortality rate is not encouraging. Our findings suggest that LCK may be a novel potential biomarker for predicting immunotherapy in melanoma. The findings show that in melanoma, patients with high LCK expression have a higher degree of immune cell infiltration *in vivo* (**Figure 5A**), which also corresponds to a higher overall survival (**Figure 5B**). LCK (lymphocyte-specific protein tyrosine kinase) belongs to the SRC family of tyrosine kinases and has been best studied in the context of T-cell function and signaling as well as lymphocytic leukemia of the B-cell lineage (14). LCK is mainly expressed in T cells, NK cells, B cells. In the present study, based on single-cell RNA sequencing technology, we observed specific expression of LCK on T cells (**Figure 6B**). In addition, a growing number of studies have shown that LCK is also expressed in brain and tumor cells, where it is actively involved in the regulation of cellular functions such as proliferation and survival (14, 34, 35). LCK is highly expressed in most cancers, including breast cancer, colorectal cancer and glioma (36–39). We found that the expression level of LCK was significantly higher in melanoma tumor tissues than in normal tissues (**Figure 5C**). Moreover, the results of RT-qPCR showed that LCK expression was significantly upregulated in melanoma cell lines compared to normal skin cell lines (**Figure 5D**). In addition, LCK was detected in the C2 subgroup of melanoma cells (**Figure 6D**).

LCK plays a vital role in various cellular processes such as cell cycle control, cell adhesion, motility, proliferation and differentiation (40). This encoded protein is a key signaling molecule for selection and maturation during T cell development (41). In recent years, it is reported that LCK has been determined as one of the key molecules regulating T cell function, and studies using knockout LCK mice or LCK-deficient T cell lines surface that LCK regulates signal initiation, T cell development and T cell homeostasis and also can enhance or inhibit BCR signaling (35, 42). Patients with LCK deficiency frequently present with immune dysregulation and autoimmunity. Overexpression of LCK contributes to a large number of other diseases such as cancer, systemic lupus erythematosus and organ transplant rejection (43). It has been reported that knockdown of LCK significantly inhibits cell proliferation and cell invasion in Oral squamous cell carcinoma (OSCC) (44). Another report stated that inhibition or downregulation of LCK led to apoptosis in Chronic Lymphocytic Leukemia (CLL) cell lines (45). Therefore, the application of LCK inhibitors could be an important strategy for the treatment of certain cancers (46). However, it has also been reported that high expression of LCK is associated with increased cumulative survival in melanoma patients (37). This is consistent with the results of our study. In an *in vivo* study of mice with LCK-deficient CLL disease model, it was found that LCK-KO group mice had a significantly shorter median survival compared to wild-type healthy mice over an observation period of 350 days (47).

LCK functions primarily in lymphocytes and is involved in transduction from the T-cell receptor complex to the nucleus, and this specific expression and function in immune cells may partially explain the phenomenon that high LCK expression is often associated with extended overall survival. Interestingly, we found that LCK in melanoma cells and T cells facilitates the interaction between the two cells by binding to the CD8 receptor. Previous studies have shown that the binding of LCK to the CD8 receptor drives CD8 close to the TCR and ultimately stabilizes the TCR-MHCp interaction, which then further promotes the activation of T cells (31, 32). The activated state of T cells enhances the responsiveness to pathogens or malignant cells, while further inhibiting tumor progression (33, 48). Therefore, since LCK plays a role in cancer cell signaling as well as in T-cell function, it will be necessary to define therapeutic strategies to selectively target LCK in tumor cells without impairing the responses of tumor infiltration lymphocytes. This is a critical issue common to other kinase inhibitors targeting signaling molecules expressed in both cancer and immune cells (e.g., BRAF, AKT, mTOR inhibitors) (35).

## Conclusion

In this study, through bioinformatic analysis of core immune escape related genes, we conclusively identified LCK as a prognostic biomarker that could remodel TME. LCK is associated with prolonged overall survival and is predictive of response to immunotherapy. In addition, LCK in melanoma cells and T cells further activates T cells by binding to CD8 receptors, promoting anti-tumor response of T cells and suppressing immune escape phenomenon. Notably, therapeutic approaches that target LCK in tumor cells may offer a new perspective for the treatment of melanoma.

## Data availability statement

The original contributions presented in the study are included in the article/**Supplementary Material**. Further inquiries can be directed to the corresponding authors.

## Author contributions

Conceptualization and methodology: FW, AZ, DZ; Formal analysis and investigation: FW, AZ, DZ, MX, JC, SX, BW; Cell experiments: TZ; Writing original Draft preparation: AZ, DZ, ZZ, XW, ML; Writing review and editing: ZX, FD, YC, YZ, QW, JL, JS, SD. All authors contributed to manuscript revision, read, and approved the submitted version.

## Funding

This work was supported by National Natural Science Foundation of China (No. 81972643, No. 82172962), Sichuan Science and Technology Project (2021YJ0201), Luxian People’s Government and Southwest Medical University Scientific and Technological Achievements Transfer and Transformation Strategic Cooperation Project (2019LXXNYKD-07) and Science and Technology Program of Luzhou, China (No. 2017LZXNYD-Z04, No. 21CGZHPT0001).

## Acknowledgments

Thanks to MX, JC, SX, BW for their help in analysis and TZ for experiments, and to ZX, ZZ, XW, ML, FD, YC, YZ, QW, JL, JS and SD for their help in writing and revision.

## Conflict of interest

The authors declare that the research was conducted in the absence of any commercial or financial relationships that could be construed as a potential conflict of interest.

## Publisher’s note

All claims expressed in this article are solely those of the authors and do not necessarily represent those of their affiliated organizations, or those of the publisher, the editors and the reviewers. Any product that may be evaluated in this article, or claim that may be made by its manufacturer, is not guaranteed or endorsed by the publisher.
