# Supplementary material for: Molecular profiling of core immune-escape genes highlights LCK as an immune-related prognostic biomarker in melanoma
Source: Front Immunol. 2022 Oct 20;13:1024931. doi: 10.3389/fimmu.2022.1024931 (PMC9630653; doi:10.3389/fimmu.2022.1024931)
Supplement: Supplementary file 1 [file DataSheet_1.pdf]

Supplementary figure

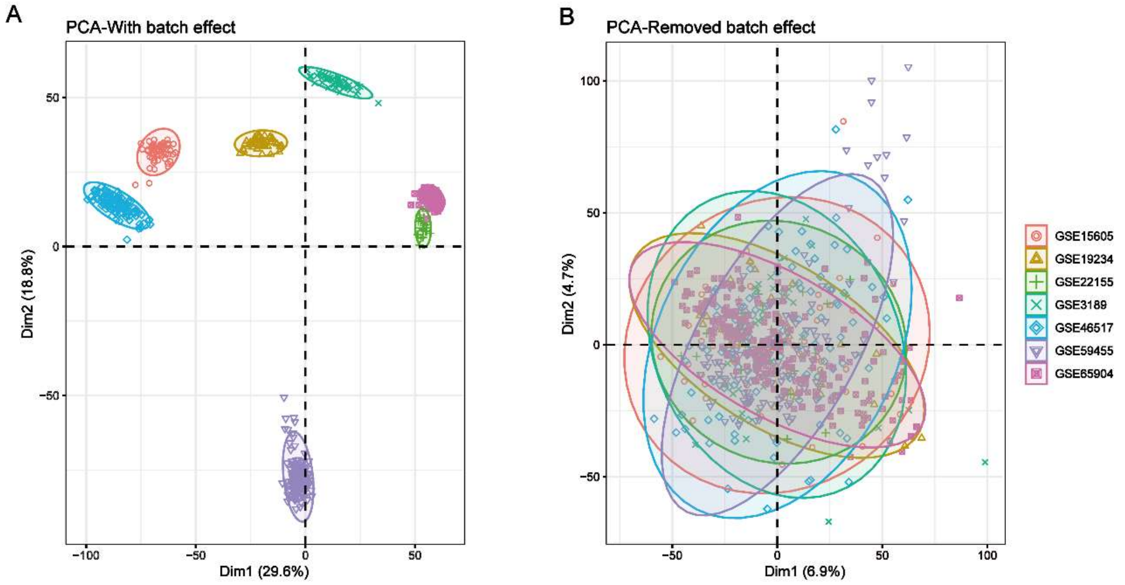

Supplementary figure 1. | Integration of GEO queues for GSEA. Different shapes in different colors represent different GEO queues.

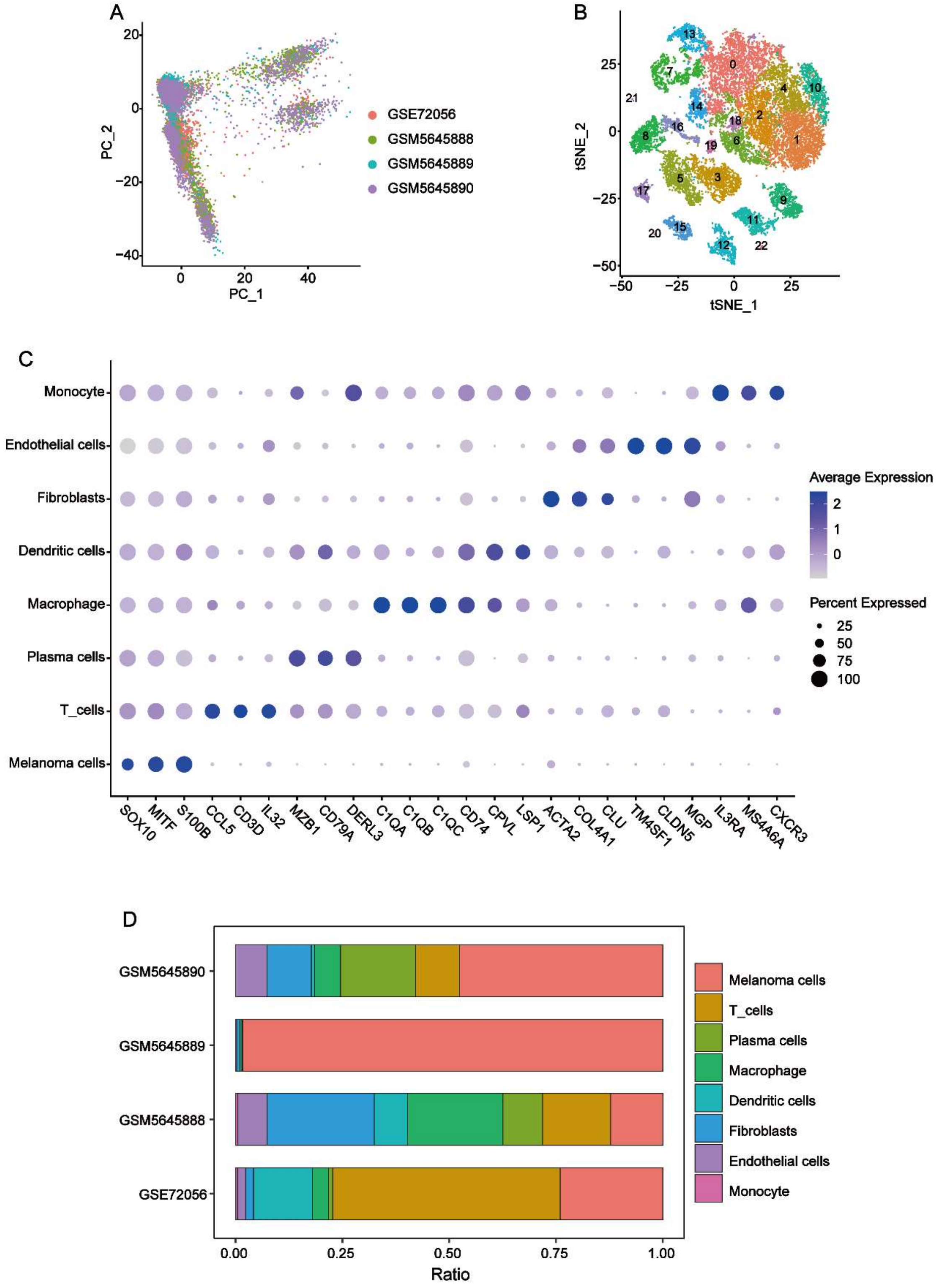

Supplementary figure 2. | (A) Integration of melanoma single-cell RNA sequencing data from GSE72066 and from GSE186344 for three samples, GSM5645888, GSM5645889 and GSM5645890, respectively. **(B)** The tSNE plot of the initial descending clustering of 18690 single cells of melanoma. **(C)** Marker gene expression for each cell type, where dot size and colour represent percentage of marker gene expression (pct. exp) and the averaged scaled expression (avg. exp. scale) value, respectively. **(D)** Cell composition distribution for each patient sample.

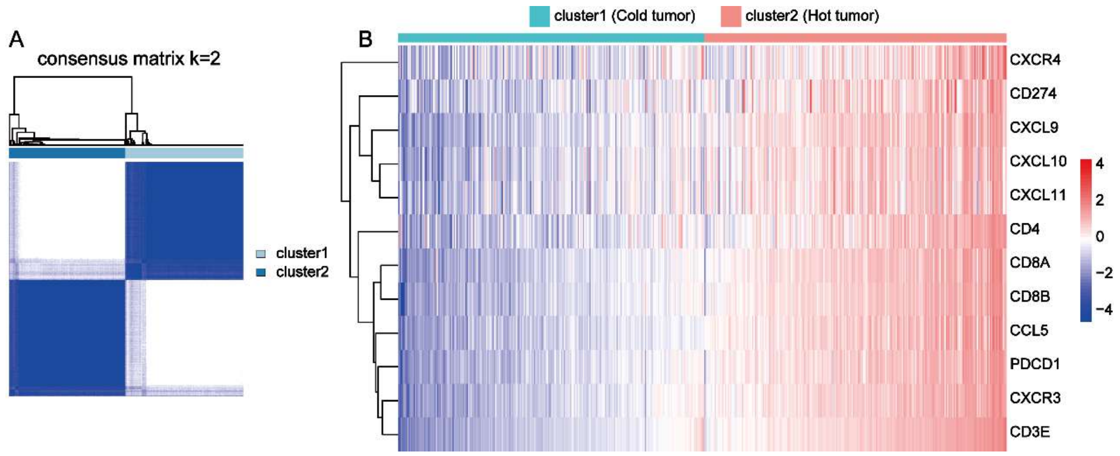

Supplementary figure 3. | (A) The consensus matrix shows the cluster members marked with blue rectangles in the condition of K=2. **(B)** Heatmap plot showed hot tumor signature genes were enriched in hot tumor samples.

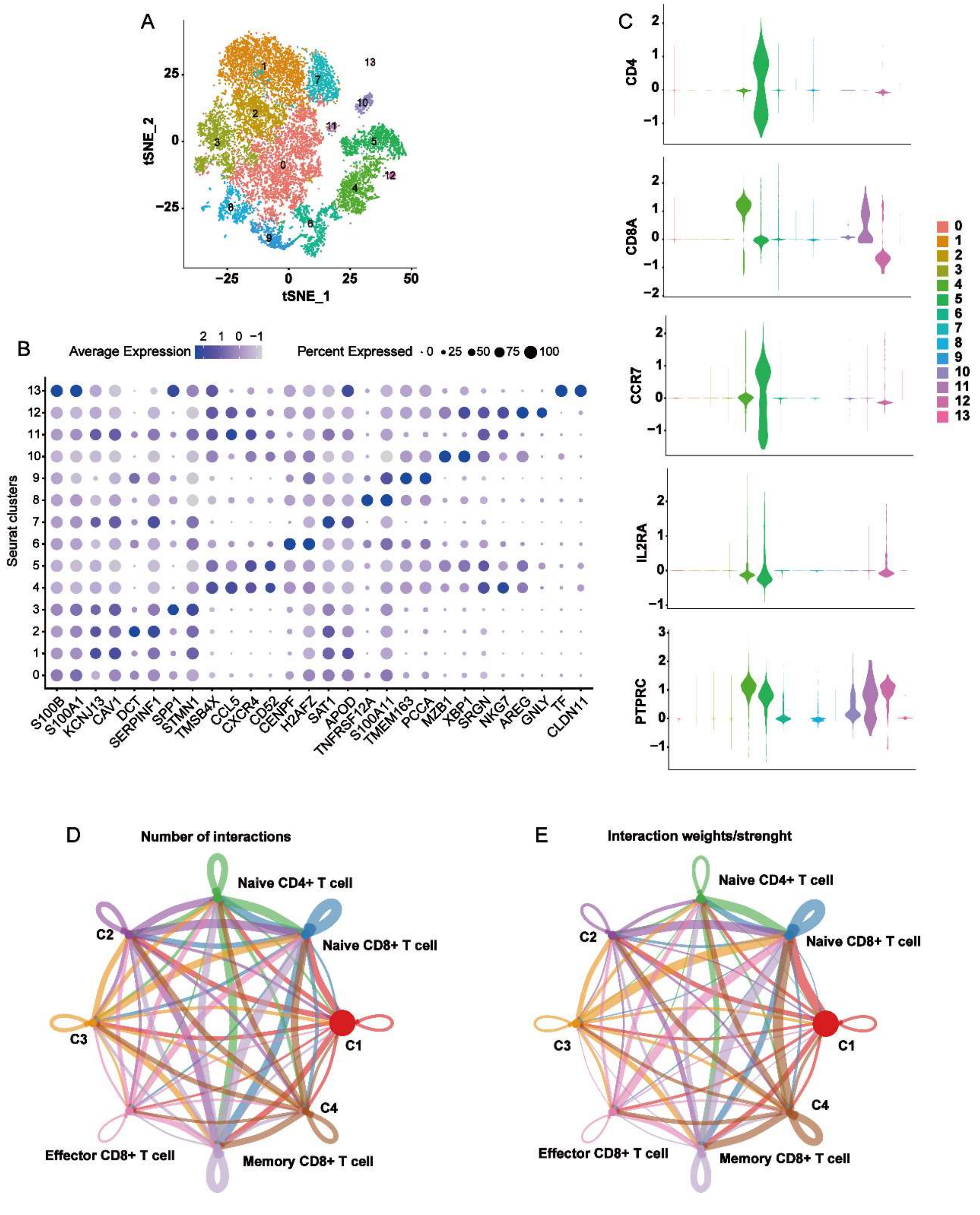

Supplementary figure 4. | (A) The tSNE plot of the initial descending clustering of 14760 single cells for melanoma cells and T cells. **(B)** Marker gene expression for each cell type, where dot size and colour represent percentage of marker gene expression (pct. exp) and the averaged scaled expression (avg. exp. scale) value, respectively. **(C)** Violin plots of markers used to identify T cell subgroups. **(D, E)** Circle plots of CellChat analysis. The left side represents the number of interactions and the right side represents the weight of the interactions. The thickness of the different colored lines represents the amount and weight of the quantity, and the arrows represent the direction.
